# Three-Dimensional Volume-Rendered Series Complements 2D Orthogonal Multidetector Computed Tomography in the Evaluation of Abnormal Spinal Curvature in Patients at a Major Cancer Center: A Retrospective Review

**DOI:** 10.5402/2012/639189

**Published:** 2012-10-04

**Authors:** J. Matthew Debnam, Leena Ketonen, Nandita Guha-Thakurta

**Affiliations:** Section of Neuroradiology, Department of Diagnostic Radiology, The University of Texas MD Anderson Cancer Center, 1515 Holcombe Boulevard, Unit 370, Houston, TX 77030, USA

## Abstract

*Background.* Abnormal spinal curvature is routinely assessed with plain radiographs, MDCT, and MRI. MDCT can provide two-dimensional (2-D) orthogonal as well as reconstructed three-dimensional volume-rendered (3-D VR) images of the spine, including the translucent display: a computer-generated image set that enables the visualization of surgical instrumentation through bony structures. We hypothesized that the 3-D VR series provides additional information beyond that of 2-D orthogonal MDCT in the evaluation of abnormal spinal curvature in patients evaluated at a major cancer center. *Methods.* The 3-D VR series, including the translucent display, was compared to 2-D orthogonal MDCT studies in patients with an abnormal spinal curvature greater than 25 degrees and scored as being not helpful (0) or helpful (1) in 3 categories: spinal curvature; bony definition; additional findings (mass lesions, fractures, and instrumentation). *Results.* In 38 of 48 (79.2%) patients assessed, the 3-D VR series were scored as helpful in 63 of 144 (43.8%) total possible categories (32 spinal curvature; 14 bony definition; 17 additional findings). *Conclusion.* Three-dimensional MDCT images, including the translucent display, are complementary to multiplanar 2-D orthogonal MCDT in the evaluation of abnormal spinal curvature in patients treated at a major cancer center.

## 1. Background

**Figure 1:**
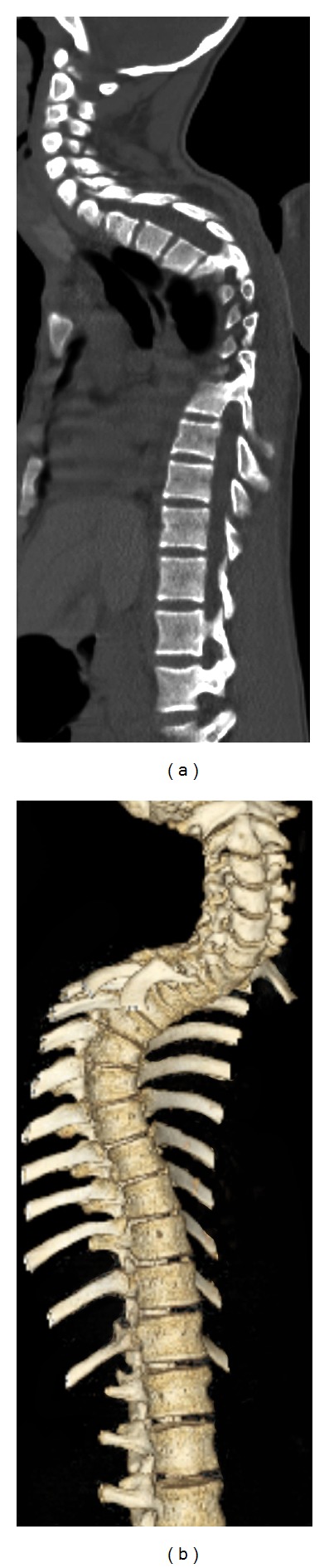
Patient 11, a 16-year-old male who underwent resection of an intramedullary juvenile pilocytic astrocytoma and presented with increasing thoracic kyphosis. (a) Sagittal MDCT image demonstrating the kyphotic deformity. Although consecutive images can be scrolled through the spine, each image is viewed singularly, limiting the evaluation of the abnormal curvature. (b) 3-D volume-rendered series, demonstrating kyphotic spinal curvature, can be rotated and viewed from 360°.

**Figure 2:**
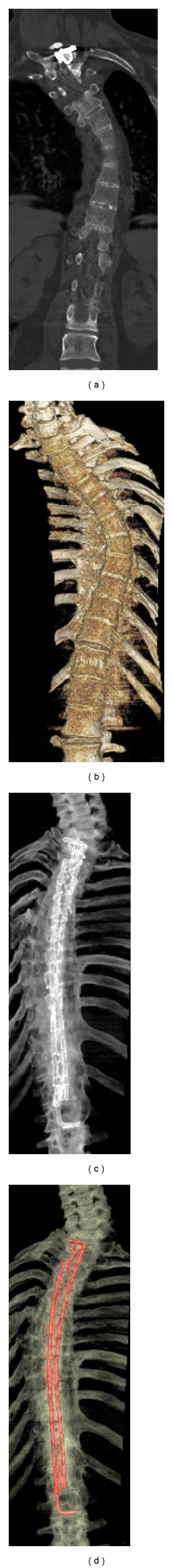
Patient 4, a 61-year-old woman with breast cancer who underwent correction of scoliosis with rod fixation at an outside institution. (a) 2-D coronal MDCT reconstruction demonstrating the scoliotic deformity and only a portion of the posterior instrumentation. (b) 3-D volume-rendered series demonstrating the surface of the vertebral bodies in the scoliotic spine. (c) 3-D volume-rendered series with transparent display demonstrating the position and integrity of the spinal instrumentation. This series can be rotated and viewed from 360°. (d) Color-coded 3-D volume-rendered series with transparent display demonstrating the spinal instrumentation same image as (c), but color-coded to illustrate instrumentation.

Abnormal spinal curvature may be idiopathic or secondary to dystrophic etiologies, such as congenital, traumatic, and malignant causes. Initial assessment and followup of patients with an abnormal spinal curvature have routinely been performed using plain radiographs [1]. To evaluate dystrophic features, magnetic resonance imaging (MRI) has also been utilized [2, 3]. Computed tomography (CT) is proving to be of benefit in the assessment of patients with an abnormal spinal curvature [4–10]. Recent advances in multidetector CT (MDCT) technique allow the evaluation of the spine in multiple 2-D planes (Figures 1(a) and 2(a)) and with a three-dimensional volume-rendered series (3-D VR) (Figures 1(b) and 2(b)). In addition, the data from the MDCT study can also be used to generate a translucent display, a computer-generated image set that provides 3-D images of the spine enabling assessment of surgical instrumentation through the bony structures (Figures 2(c) and 2(d)). The 3-D VR series, including the translucent display, which are generated by computer manipulation of the axial CT source data without additional radiation, have led to a growth in demand for MDCT for the imaging of the spine by the spine surgeons at our institution, a major cancer center. However, to the best of our knowledge, there are no reports in the literature on the use of MDCT with the 3-D VR series and the translucent display for evaluation of abnormal spinal curvature, which requires additional time for processing and interpretation. We tested the hypothesis that the 3-D VR series, including the translucent display, provides additional information beyond that of the 2-D orthogonal MDCT in the evaluation of abnormal spinal curvature in patients treated at a cancer center. 

## 2. Methods

The Institutional Review Board of The University of Texas MD Anderson Cancer Center approved this study and waived the requirements for an informed consent. MDCT studies of the spine in patients who had a history of possible primary or metastatic disease to the spine or neurofibromatosis type 1 and an abnormal spinal curvature greater than 25 degrees, as assessed on MDCT, were included in our study. Measurements of spinal curvature were made on the 2-D coronal or sagittal reconstructed CT images, as described in the literature [4, 5]. The review of the imaging studies and clinical data was performed by 3 neuroradiologists (JMD, LK, and NGT) in a consensus fashion. The 3-D VR series, including translucent display, was compared to the 2-D orthogonal MDCT studies without the 3-D VR series in the assessment of the following 3 categories: spinal curvature; bony definition (deformity, fusion, and destruction); additional findings (mass lesions, fractures, and instrumentation). For each patient, the 3-D VR series was given a score of 1 if it was judged to be “helpful” in the evaluation of the aforementioned 3 categories (48 patients × 3 categories = 144 total possible categories) and a score of 0 if it was “not helpful”.

MDCT examinations were performed on a multidetector CT scanner (GE Medical Systems) to yield imaging in the axial plane using the following parameters: 140 kV, 220–250 mA, and a 1.25 mm slice thickness. The MDCT examinations were performed without (*n* = 41), with (*n* = 3), or without and with (*n* = 4) intravenous contrast (Optiray, Mallinckrodt Inc., St. Louis, MO, USA). Bone algorithm and soft tissue images were available and reviewed in all patients' MDCT studies. Postprocessing was then performed by a trained technologist on an advantage AW4.2 workstation (GE Medical Systems) using Volume View software (GE Medical Systems). The post-processing provided imaging in the sagittal and coronal planes in all patients, and these images, together with the axial source images, are hereafter referred to as the 2-D orthogonal MDCT study. In addition, 3-D VR images of the spine (Figures 1(b) and 2(b)) and the translucent display (Figures 2(c) and 2(d)), for patients in whom surgical instrumentation was placed for the stabilization of abnormal spinal curvature, were provided. 

## 3. Results

The study included 48 patients (35 female and 13 male; ages 12–72, mean age, 48 years), as summarized in Table 1. Twenty-four patients had a dextroscoliotic curvature measuring between 26 and 93 degrees (mean, 45.5), 9 patients had a levoscoliotic curvature measuring between 25 and 72 degrees (mean, 35.2 degrees), and 15 patients had a kyphotic curvature of measuring between 27 and 89 degrees (mean 48.1 degrees). Twenty-six patients had a 3-D VR series without a transparent display and 22 patients had a 3-D VR series with a transparent display. 

The 3-D VR series was rated as “helpful” when compared to the 2-D orthogonal MDCT study in 38 of 48 (79.2%) patients; in 10 of 48 (20.8%) patients, the 3-D VR series was rated as “not helpful”. The 3-D VR series were scored as “helpful” in 63 of 144 (43.8%) total possible categories (Table 2). This included the assessment of the spinal curvature in 32 of 48 (66.7%) patients, including dextroscoliosis (*n* = 16, 26−83°, mean 50.6°), levoscoliosis (*n* = 8, 25–72°, mean 35.3°), and kyphosis (*n* = 8; 42–89°, mean 58.9°). The 3-D VR series was rated as “not helpful” in 16 of 48 patients (33.3%), including those with dextroscoliosis (*n* = 8; 29–49°, mean 35.4°), levoscoliosis (*n* = 1, 35°), and kyphosis (*n* = 7; 27–47°, mean 35.7°). 

The 3-D VR series was rated as “helpful” in the bone definition category for 14 of 48 (29.2%) patients, including bone deformity (*n* = 6), bony fusion (*n* = 4), bony destruction (*n* = 2), anterolisthesis (*n* = 2), and the additional findings category, including surgical instrumentation, in 17 patients, specifically, for the assessment of the fusion rods (*n* = 15) or an anterior fusion plate and pedicular screws (*n* = 2). 

## 4. Illustrative Cases

Figure 1 shows 2-D orthogonal MDCT and 3-D VR images from patient no. 11, a 16-year-old male who presented with a kyphotic curvature secondary to a recurrent juvenile pilocytic astrocytoma of the thoracic spinal cord. The 3-D VR images were scored as 1, “helpful” in the evaluation of the spinal curvature as the spine is out of plane on 2-D orthogonal MDCT imaging.

Figure 2 shows 2-D orthogonal MDCT and 3-D VR images from patient no. 4, a 61-year-old woman who underwent correction of an abnormal spinal curvature at an outside institution. The patient had a levoscoliotic curvature of the thoracic spine to such a degree the spinal column and instrumentation could not be visualized as one structure on a single, 2-D orthogonal MDCT image. The transparent display was helpful in demonstrating the scoliotic curvature and the position and integrity of the instrumentation.

## 5. Discussion

The 3-D VR series was rated as more “helpful” in comparison to the corresponding 2-D orthogonal MDCT in the assessment of abnormal spinal curvature in 38 of 48 patients, specifically in the evaluation of abnormal curvature in 32 patients, bony definition in 14 patients, and additional findings, including surgical instrumentation, in 17 patients. These findings confirm our hypothesis that the 3-D VR series, including the translucent display, is of additional benefit in the assessment of abnormal spinal curvature in patients treated at a major cancer center. As not all patients had abnormalities in each of the 3 categories, that is, surgical instrumentation, the percentage of cases where the 3-D VR was helpful is likely higher than what we report, further supporting our hypothesis. 

When reviewing an MDCT study of abnormal spinal curvature, the 3-D VR series can be assessed before the 2-D orthogonal MDCT study. As the spinal deformity in these patients is often out of the plane of imaging on a single, 2-D orthogonal MDCT image (Figures 1(a) and 2(a)), the 3-D VR sequence provides comprehensive assessment of the entire spine on a single 3-D image; this image can then be rotated and viewed from 360 degrees. In our study, we found this more beneficial in patients with a greater degree of dextroscoliotic than kyphotic curvature and more helpful for a greater degree of abnormal spinal curvature. Not only can the 3-D VR series evaluate the shape of the spine, but also aid in the detection of a rotatory component, anterolisthesis, and the apex of the curvature. Any detected findings can then be confirmed on the 2-D MDCT study. In addition, with the 3-D VR series, the number of vertebral bodies can be counted in a single view; this assures that the numbering assignment will be correct if an anomalous number of spinal segments are present. This is more difficult to determine on orthogonal 2-D MDCT as the spine is often out of the plane of imaging due to the spinal curvature.

The 3-D VR series can also evaluate the vertebral bodies for evidence of dysplasia, fracture, or bony destruction. Previous authors [10–12] have demonstrated that CT is better than plain radiographs for the evaluation of spine abnormalities. Kim et al. [10] state that when a complex osseous deformity is present, radiographs are inadequate for complete evaluation and the use of CT is mandatory, especially when surgery is planned. Our result takes this evaluation as a step further and demonstrate that the 3-D VR series would benefit 2-D orthogonal MDCT in the evaluation of vertebral anomalies as the 14 studies scored a “helpful” rating in the evaluation of the bony structures, including 4 cases with prior surgical bony fusion. 

In the post-operative patient, the 3-D VR series with translucent display may be used to assess surgical instrumentation [13]. This technique allows the visualization of the instrumentation through the bone and can also be rotated and viewed in 360 degrees, including in oblique planes. As the surgical instrumentation is visualized as one component on a single image, and the position and integrity of support rods and pedicular screws are assessed, coloring of the spinal instrumentation is also possible (Figure 2(d)). In every case with surgical instrumentation in our study, the 3-D VR with translucent display was rated as “helpful”. In fact, our referring spine surgeons insist on inclusion of the 3-D VR series for the evaluation of the spine and the transparent display for a comprehensive overview of the surgical instrumentation. Further study can be undertaken to determine if the 3-D VR series with translucent display can be used to evaluate surgical instrumentation following spinal surgery in the general population with an abnormal spinal curvature and to evaluate associated complications.

As recent advances in MDCT technology have led to a significant reduction in streak artifact related to metallic hardware [13, 14]. MDCT with VR series may lead to better evaluation of the postoperative spine and possibly easier detection of complications. It should be noted, though, that the interpreting radiologist must be careful not to mistake streak artifact that extends through the surgical instrumentation of the breakage of hardware. 

The purpose of this study was to determine if the 3-D VR images provide additional information to the orthogonal 2-D MDCT dataset and the results support our hypothesis. We are not suggesting that 3-D VR can replace the orthogonal 2-D MDCT; rather 3-D VR is complementary. One limitation of the study is that this is a very select group of patients, mainly those presenting for treatment of their disease to a major cancer center. 

Computed tomography has been described in the measurement of scoliosis, including the rotatory component [15–19]. Further study will be necessary to determine if the 3-D VR series can be for evaluation and measurement of abnormal curvature in the general population, including for idiopathic scoliosis. One negative aspect of MDCT is that patients are imaged in the recumbent position and the use of ionizing radiation. The downside of patient positioning also applies to the reconstructed 3-D VR series; however, no addition radiation is necessary for computer generation of the 3-D VR series or the translucent display. 

## 6. Conclusion

Herein, we have illustrated the added benefit of 3-D VR imaging and the translucent display to axial and 2-D orthogonal MDCT imaging of the spine for the evaluation of abnormal spinal curvature among patients at a major cancer center. Failure to recognize the etiology of spinal curvature, such as syndromic deformities, fractures, or malignancies of the spine, can affect treatment and management outcomes for patients. The translucent display series provides a more comprehensive evaluation of surgical instrumentation following correction of an abnormal spinal curvature or resection of malignancy. It is, therefore, beneficial for spine surgeons and radiologists involved in the care of patients with abnormal spinal curvature to be aware of the benefits of the 3-D VR series and the translucent display.

## Figures and Tables

**Table 1:** Patient demographics.

Patient no.	Age/sex	Reason for MDCT	Curvature (degrees)	Type
1	18/F	NF-1	81	d
2	32/F	NF-1	46	k
3	63/F	NF-1	44	k
4	61/F	Breast cancer	30	d
5	31/F	Undiagnosed paraspinal mass	69	d
6	38/F	NF-1	57	k
7	36/F	NF-1	72	l
8	44/M	NF-1	56	k
9	15/F	Histiocytosis	35	d
10	63/F	Thyroid cancer	61	k
11	16/M	JPA	76	k
12	53/F	Breast cancer	35	d
13	12/M	NF-1	50	d
14	42/F	NF-1	39	d
15	20/F	NF-1	57	d
16	19/F	NF-1	33	d
17	19/F	NF-1	30	d
18	29/F	NF-1	52	d
19	65/F	Multiple myeloma	42	k
20	68/F	Pyriform sinus SCC	29	k
21	77/M	Lung cancer	29	k
22	54/F	Breast cancer	35	d
23	61/M	Multiple myeloma	49	d
24	21/M	Pelvic Ewings sarcoma	40	l
25	53/M	Thyroid cancer	35	l
26	66/F	Undifferentiated sarcoma of the spine	42	k
27	44/F	NF-1	54	d
28	58/F	Breast cancer	39	k
29	56/F	NF-1	55	d
30	32/F	NF-1	46	d
31	82/M	Larynx chondrosarcoma	47	k
32	59/F	Met myxoid liposarcoma pelvis	37	k
33	61/M	Desmoid s/p laminectomy	30	l
34	77/F	Multiple myeloma	27	l
35	75/M	Prostate cancer	26	d
36	30/F	NF-1	41	d
37	39/F	NF-1	93	d
38	40/F	Cervical cancer	36	l
39	62/F	Breast cancer	27	k
40	67/F	Thyroid cancer	27	l
41	71/F	MFH	29	d
42	56/F	NF-1	53	d
43	55/M	Lymphoma	34	d
44	66/F	L3 plasmacytoma	37	d
45	33/F	Residual ependymoma	25	l
46	44/F	Lung cancer	30	d
47	71/M	Esophageal cancer	89	k
48	50/M	HCC	25	l

NF-1: neurofibromatosis type 1,

JPA: juvenile pilocytic astrocytoma,

SCC: squamous cell carcinoma,

MFH: malignant fibrous histiocytoma,

d: dextroscoliosis,

l: levoscoliosis,

k: kyphosis.

**Table 2:** Categories—MDCT/3-D VR helpful to MDCT.

Patient no.	Category
1	Curvature, surgical fusion, and fusion rods
2	Curvature, anterolisthesis
3	Curvature
4	Curvature, fusion rods
5	Curvature, fusion rods
6	Curvature, fusion rods
7	Curvature, deformity, and fusion rods
8	Curvature, fusion rods
10	Curvature, anterolisthesis
11	Curvature
12	Destruction
13	Curvature
14	Curvature
15	Curvature, deformity
16	Fusion rods
17	Fusion rods
18	Curvature, fusion rods
20	Bone plate/screws
23	Pedicle screws
24	Curvature, destruction
26	Curvature, fusion rods
27	Curvature, surgical fusion, and fusion rods
29	Curvature, surgical fusion, and fusion rods
30	Curvature, deformity
32	Surgical fusion
33	Curvature
34	Curvature
35	Curvature
36	Curvature, deformity
37	Curvature, deformity, and fusion rods
38	Curvature
40	Curvature
42	Curvature, deformity, and fusion rods
43	Curvature
45	Curvature, fusion rods
46	Curvature
47	Curvature
48	Curvature

## Abbreviations

MRI: Magnetic resonance imaging
CT: Computed tomography
MDCT: Multidetector CT
2-D: Two-dimensional
3-D VR: Three-dimensional volumerendered.
